# Sexual Dysfunction and Quality of Life in Patients with Rheumatoid Arthritis

**DOI:** 10.3390/ijerph19053088

**Published:** 2022-03-06

**Authors:** Wojciech Tański, Krzysztof Dudek, Anna Tomasiewicz, Natalia Świątoniowska-Lonc

**Affiliations:** 1 Department of Internal Medicine, 4th Military Teaching Hospital, 50-981 Wroclaw, Poland; 2 Department of Logistic and Transportation Systems, Wroclaw University of Science and Technology, 50-370 Wroclaw, Poland; krzysztof.dudek@pwr.edu.pl; 3 Student Research Club of Surgical Specialties, Faculty of Medicine, Wroclaw Medical University, 50-367 Wroclaw, Poland; anna.tomasiewicz@student.umw.edu.pl; 4 Center for Research and Innovation, 4th Military Teaching Hospital, 50-981 Wroclaw, Poland; natalia.swiat@o2.pl

**Keywords:** sexual dysfunctions, quality of life, rheumatoid arthritis

## Abstract

Background: Sexual health is a major component of human well-being. As repeatedly shown in research, satisfaction with sex life and sexual fulfillment correlate positively with quality of life (QoL) in most of its aspects. It is thus true that a reduced quality of one’s sex life and lack of sexual fulfillment can contribute to poorer QoL overall. The aim of this study is to describe an assessment of sexual dysfunction and factors affecting sexual dysfunctions of patients with rheumatoid arthritis (RA). Material and methods: 171 consecutive RA patients (mean age 48.3 ± 14.6) attending the rheumatology outpatient clinic. Standardized questionnaires used in the study were the sexological questionnaire, WHOQOL-BREF to assess QoL level, Disease Acceptance Scale, and VAS scale to assess pain intensity. Results: The mean duration of the disease in the study group was 13 ± 9 years, mean score of subjective assessment of mobility was 6.2 ± 1.6, and the mean score of the DAS-28 was 4.0 ± 1.9. The study group presented a mean level of disease acceptance (AIS 29.6 ± 11.6). The comparative analysis showed significant differences in reaching orgasm and declared sexual dysfunctions. These problems occurred more often in women than in men (34.2% vs. 18% and 43% vs. 40%, respectively). In univariate analysis, factors correlating positively with the frequency of declaring sexual dysfunction were subjective motor score less < 6 points, AIS < 36 points, WHOQOL-BREF < 59 points, disease activity ≥3.5 points, and VAS > 3. In multivariate logistic regression analysis, independent factors positively correlating with frequency of sexual dysfunction declaration were general QoL (β = 1.255; *p* = 0.035) and pain limiting social life (β = 1.564; *p* = 0.030). The absence of comorbidities correlated negatively and reduced the prevalence of sexual dysfunction (β = −1.030; *p* = 0.043). Patients with reduced QoL and patients with pain limiting social life had 3.5 and 4.8 times higher risk of sexual dysfunction than other patients, respectively. In contrast, those without comorbidities were 2.8 times more likely to be free of sexual dysfunction than those diagnosed with other chronic diseases besides RA. Conclusions: Sexual dysfunction is an emerging problem in both men and women with RA. The absence of comorbidities is an independent determinant of sexual dysfunction, whereas poor QoL and pain limiting social life are independent determinants that exacerbate sexual dysfunction in both genders.

## 1. Background

Rheumatoid arthritis (RA) is characterized by immune-mediated destruction of the joints. Epidemiological studies show that the disease affects 0.5–1% of the adult population and is 2–3 times more common in women [1]. It often leads to joint damage, impaired mobility, pain, and fatigue. RA typically affects the joints, but it can also involve the internal organs and cause serious multiple-organ complications, even leading to premature mortality. Without early diagnosis and treatment, the disease may result in progressive disability and damage to multiple organs [2]. Limitations associated with the disease, both physical and psychological, may affect patients’ sex lives [3]. RA can affect sexual function in various ways, including pain, joint stiffness and swelling, chronic fatigue, and limitations in activities of daily living [4]. The very fact that the disease is chronic and requires long-term treatment, as well as the adverse effects of RA medication, may also cause sexual dysfunctions in this patient group [5]. Sexual health is a major component of human well-being [6]. The World Health Organization (WHO) defines sexual health as “a state of physical, mental, emotional, and social well-being in relation to sexuality” [6]. Sexuality is a very important, inherent part of human functioning. As repeatedly shown in research, satisfaction with sex life and sexual fulfillment correlate positively with quality of life (QoL) in most of its aspects [7]. It is thus true that a reduced quality of one’s sex life and lack of sexual fulfillment can contribute to poorer QoL overall [8]. As poor sex life quality and sexual dissatisfaction result from sexual dysfunction, effective treatment of such a dysfunction can be assumed to contribute significantly to a better QoL [7].

Sexuality is an innate and natural dimension of human functioning, manifesting as sexual need or desire, the associated physiologically determined sexual responses, and behaviors leading to orgasm or at least to pleasurable arousal, often undertaken between two individuals but in many cases also performed alone [9]. It is also a source of joy and satisfaction, a driving force for personal growth, and one of the main motivators to establish bonds and engage in interpersonal relationships. The sexual physiological potential in humans is shaped by life experiences, and chronic illness is one such experience that may affect it considerably. Sexual health should be subject to the same kind of evaluation in daily clinical practice as the patients’ physical and psychological function. According to the Diagnostic and Statistical Manual for Mental Disorders (5th edition, 2013), sexual dysfunction entails the following disorders: delayed ejaculation, erectile disorder, female orgasmic disorder, female sexual interest/arousal disorder, genito-pelvic pain/penetration disorder, male hypoactive sexual desire disorder premature (early) ejaculation, substance/medication-induced sexual dysfunction, other specified sexual dysfunction, and unspecified sexual dysfunction [10]. Sexual dysfunction is currently becoming an increasingly widespread medical problem in middle-aged and elderly men and women, with epidemiological estimates placing its prevalence at around 18.4–30% in men and around 25.8–67% in women with RA [11]. The most common sexual dysfunctions include reduced sex drive and premature ejaculation in men, and reduced desire and arousal, difficulty in reaching an orgasm, as well as pain and discomfort during intercourse in women [12]. The causes of sexual dysfunctions are typically complex, involving a number of physical, psychological, and interpersonal determinants. They may cause severe personal distress and relationship problems. In addition, when analyzing sexual health issues in chronically ill patients, one must not forget the adverse impact of age, diabetes, cancer, cardiovascular disease, dyslipidemia, and depression, all of which may also concern patients with RA. The topic of sexual dysfunctions in RA has not yet been comprehensively investigated, but evidence does demonstrate that it is commonly diagnosed in this patient group, affecting as many as 37–66% of them [13]. Research shows that 56% of RA patients experience problems having sexual intercourse due to the pain and fatigue associated with the disease [13,14]. Available studies demonstrate that individuals who cannot reach orgasm or experience satisfaction during sexual intercourse are less likely to attempt it [13,15]. Sadly, data on any associations between sexual dysfunctions and RA, as well as any contributing or protective factors, are not yet well understood and require confirmation in further studies. The few publications available emphasize the fact that sexual dysfunctions affect both sexes in this patient group, but are more common in women than in men with RA [16,17,18]. Authors report that the sexual dysfunctions experienced by women with RA not only aggravate their physical discomfort, but also their psychological distress, and affect their social and family life, disrupting their relationships [16]. The loss of intimacy during illness causes most patients to suffer in silence and in isolation, as the issue is considered “shameful”. The health problems arising from the underlying disease, combined with the associated sexual dysfunctions, strongly affect patients’ perceived QoL, which is a significant measure complementing clinical data in holistic patient care. So far, few studies have investigated sexual dysfunctions in RA patients and their association with QoL. In light of the above, the main purpose of the present study is: (1) to describe an assessment of sexual dysfunctions among men and women suffering from RA as part of a patient-reported outcome measures assessment, (2) to identify the underlying problems in sexual life, (3) to investigate associations of disease activity and other parameters (clinical: time from RA diagnosis, comorbidities, and pain; psychological: acceptance of illness; and demographic: age and level of education) with sexual function, and (4) to assess the correlation between sexual dysfunctions and QoL.

Study hypotheses:

(1) There are gender differences in SD; women with RA have more SD than men.
(2) Pain and functional status affect the incidence with SD.
(3) Disease activity and illness duration increase the risk of SD. Disease acceptance and absence of comorbidities decrease the risk of SD.
(4) Quality of life has a positive impact on sexual functions.

## 2. Methods

### 2.1. Procedure

The data were obtained from an observational cross-sectional study, which included 171 consecutive RA patients consulted at an outpatient rheumatology clinic from January to September 2021. The inclusion criteria were the ability to meet the American College of Rheumatology diagnostic criteria for RA.

All patients were asked to complete their survey on their own, either during the visit or in the waiting room. A rheumatologist evaluated the patients’ clinical condition, and a nurse was present in case a patient needed assistance. The main objective was to obtain questionnaires that the patients completed themselves. Clinical data were obtained from the patients’ medical documentation.

The patients were informed about the purpose and course of the study and the fact that they could withdraw from the study at any time. The study was voluntary and anonymous. Patients were free to refuse or drop out during the study without giving any reason. The study was approved by the relevant Bioethics Committee, at the Military Medical Institute in Warsaw (approval no. 170/2020; date: 10.05.2020).

### 2.2. Study Instruments

The sexological questionnaire developed by Andrzej Kokoszka is used for the self-assessment of sexual dysfunction prevalence according to the International Classification of Diseases (ICD-10) criteria. The instrument comprises 30 items. Part A is used to obtain basic sociodemographic data. Part B includes 12 items on sexual dysfunction, 2 questions pertaining to the symptoms of gender dysphoria, 13 questions related to the symptoms of paraphilias, and 3 questions on sexual orientation. Respondents provide answers ranging from “always”, through “often”, to “never.” The questions may cover any period of time, depending on the specifications of a particular study. Sexual dysfunction is a subclass of sexual dysfunctions (alongside disorders of sexual preference and gender identity disorder) involving abnormal sexual response in women and men. The sexological questionnaire is a good screening tool and has good specificity and sensitivity (sexual dysfunction: 98%, 48%; gender identity disorder: 100%, 100%; sexual orientation disorders: 100%, 92%; and abnormal sexual preferences: 62%, 79%, respectively) [19].

The WHOQOL-BREF quality of life questionnaire is a generic questionnaire consisting of 26 items and measuring six QoL domains: overall perceived QoL, perceived general health, physical health, psychological, social relationships, and environment. The measurement covers the preceding 14 days. Item scores in each domain are summed up and converted into a scale ranging from 0 to 100. The higher the score, the better the respondent’s health-related quality of life (HRQoL). The WHOQOL-BREF presents good internal consistency, sensitivity to change, and discriminant validity. In the original version of the questionnaire, the Cronbach’s alpha coefficient for the entire scale was 0.896. The internal reliability for all domains was >0.70, except for the domain “Social Relationships” (0.533). This means that it is an excellent instrument to discriminate between ill and healthy subjects [13]. The Polish version of the WHOQoL-BREF has good psychometric properties (Cronbach’s α for the physical domain—0.81, psychological—0.78, social—0.69, for the environment—0.77, and for the whole questionnaire—0.90) [20].

The Acceptance of Illness Scale (AIS) is a standardized research instrument developed by Felton et al. and adapted into Polish by Juczyński [21]. It is used for assessing patients’ acceptance in various illnesses. The scale consists of eight statements pertaining to the limitations and difficulties caused by the illness: sense of being dependent on others, lack of independence, and decreased self-esteem. Respondents provide answers on a 5-item Likert scale. The scores range from 8 to 40 points [22]. Scores of >30 reflect high acceptance, 19–29 reflect moderate acceptance, and 8–18 reflect low acceptance of illness. The internal consistency and reliability of the Polish version of the scale (Cronbach’s alpha = 0.82) are similar to the values obtained for the original AIS [23].

The Visual Analog Scale (VAS) is a reliable tool for pain severity assessment. Regularly repeated measurements allow for assessing the effectiveness of any analgesic treatment administered. The VAS is a 10-cm line, where 0 represents no pain and 10 denotes the worst pain imaginable. With a good repeatability of measurements, considerable reliability, and clarity to most patients, the scale is the most commonly used pain evaluation instrument [24]. In the original version of VAS, test–retest reliability has been shown to be good, but higher among literate (r = 0.94) than illiterate rheumatology patients (r = 0.71). The pain VAS has been shown to be highly correlated with a 5-point verbal descriptive scale and a numeric rating scale (NRS) with correlations ranging from 0.71 to 0.78 and 0.62 to 0.91, respectively [25].

The 28-joint disease activity score (DAS-28) consists of the number of swollen and painful joints among 28 subjects, as well as the erythrocyte sedimentation reaction score and the assessment of general health status on a visual rating scale. For both parameters, the health score on the VAS is optional (i.e., both DAS and DAS-28 can be calculated without it). The DAS score ranges from 0 to 10, and the DAS-28 score ranges from 0 to 9.4, so they cannot be directly compared to each other. Systematically determined DAS (DAS-28) allows the selection of patients with high risk of radiological progression of disease and disability. Cronbach’s alpha for the DAS28 in RA patients amounted to 0.7329, indicating high internal consistency [26].

### 2.3. Statistical Methods

Statistical analyses were performed using the STATISTICA v. 13.3 software (TIBCO Software Inc., Palo Alto, Ca, USA). For quantitative variables, means (M), standard deviations (SD), median (Me), lower quartile (Q1), upper quartile (Q3), and extreme (Min and Max) values were calculated. Empirical distribution fit to the Gaussian distribution for quantitative variables was verified using the Kolmogorov–Smirnov and Shapiro–Wilk tests. Qualitative (nominal and ordinal) variables were reported in contingency tables as numbers (n) and percentages (%). Continuous variables were converted into dichotomous variables using cutoff values determined by ROC curve analysis. The significance of differences in quantitative parameters between the two groups was verified using the Mann–Whitney U-test, and the independence of two qualitative parameters was verified using Pearson’s chi-squared test. For all statistical tests, a significance threshold of *p* < 0.05 was used.

## 3. Results

### 3.1. Participants’ Characteristics

A total of 194 people were invited to participate in the study who were scheduled to visit the clinic at that time. However, 17 people refused to participate before taking the survey, believing that the problem did not concern them, and 6 people returned questionnaires incomplete despite giving their consent to the study. The survey included 171 patients (mean age 48.3 ± 14.6) treated for RA. The sample included a slightly higher proportion of female patients. Most respondents had completed high school or college/university education and were professionally active. More than half of the respondents reported a good or average financial standing. The general characteristics of the patients are shown in Table 1.

The mean duration of illness in the group was 13 ± 9 years. To evaluate the severity of symptoms and limitations associated with the disease, a 10-point scale was used. The score for subjective assessment of mobility was 6.2 ± 1.6 (Table 1). Disease activity score (DAS-28) was 4.0 ± 1.9. More than half of the respondents had no comorbidities (63.1%). Some respondents reported having two (26.9%) or even three (8.2%) comorbidities. The acceptance of illness assessment in the group showed that the respondents had a moderate-to-high level of acceptance (29.6 ± 11.6). Most respondents (65.5%) had a high level of RA acceptance, but some (25.5%) did not accept their condition (Table 1).

In line with the premise of our study, to describe an assessment of sexual dysfunctions among men and women suffering from RA, further analyses included a breakdown by sex. Questionnaire results show that nearly all respondents lived with their partners, while 80% of women and 81.6% of men were sexually active (ns).

Among the 171 patients included, 80.7% of the respondents considered that they had a sex life in the last 6 months. Consequently, further questionnaire questions focused on these individuals (Table 2). Analyses of the sexological questionnaire showed no statistically significant differences between women and men, with the exception of two items. One concerned absence of orgasm, which was more common in women than in men (34.2% vs. 18%) (Table 2). The other concerned the prevalence of the reported sexual dysfunctions, which was 43% in women vs. 40% in men (*p* = 0.002). A total of 13.1% of women and 10.6% of men experienced sexual dysfunction “often”. The mean score for sexual dysfunction was very similar in both sexes: 3.4 ± 0.5 vs. 3.3 ± 0.5 (ns).

Analysis of the sex-specific items of the sexological questionnaire showed that men most commonly had problems with achieving an erection before intercourse (always—27.9%, often—14.8%), maintaining an erection during intercourse (always—24.6%, often—9.8%), and premature ejaculation (20%). Women reported vaginal dryness (54.1%—sometimes), and vaginal spasm preventing intercourse or causing pain during intercourse (13.1%—often).

Most respondents (80.7%) declared having a partner recently, and 97.1% of those respondents lived with their partner. The results of the Chi-squared and Fisher’s exact tests of independence showed that in the group that was studied, these percentages did not depend on a respondent’s sex (*p* > 0.05). All further items on the sexological questionnaire only applied to those respondents who had had a partner in the previous 6 months (*n* = 138).

There was no association between the reported gender identity disorders and the sex of the studied RA patients (*p* = 0.653). The patients’ sex was also unrelated to the incidence of sexual preference disorders (*p* = 0.590) or disorders associated with sexual orientation (*p* = 1.000) (Table 2).

### 3.2. Analysis of QoL and Acceptance of Illness in RA Patients by Sex

A comparative analysis of QoL in terms of the overall QoL and general health scales of the WHOQoL-BREF, as well as the specific WHOQoL domains, demonstrated no significant differences between the men and women with RA who were studied. Likewise, acceptance of illness did not significantly differ between the sexes. Among both female and male respondents, approximately half had a high level of illness acceptance. In terms of the Disease Activity Score-28, no significant differences were found, and nearly half of the respondents had a low disease activity (Table 3).

### 3.3. Single-Factor Analysis of the Impact of Selected Variables, Broken down by Reported Sexual Dysfunction

Comparisons of responses to the sexological questionnaire showed no statistically significant differences between the sexes, with the exception of the frequency of symptoms of sexual dysfunction. This is why in further analyses, the frequency of reported symptoms was considered. Patients were broken down into two groups: group 1—frequently reported sexual dysfunction, and group 2—rarely reported sexual dysfunction. Items to which all patients responded “never”, i.e., B19–B30, were excluded from comparisons.

In the single-factor analysis, factors positively correlated with the reported frequency of sexual dysfunctions were subjective assessment of mobility below 6 points, RA acceptance below 26 points, WHOQoL score below 59 points, and disease activity of 3.5 or higher (Table 4). The frequency of sexual dysfunction was also positively correlated with pain (scored on the VAS) that limited the patient’s ability to move (≥3), limited their social life (≥4), and affected sleeping more than 6 h per night (Table 5). Independent determinants were identified by multivariate logistic regression analysis, with the results listed in Table 6.

In the multivariate logistic regression analysis, independent positive predictors of reported frequency of sexual dysfunction were WHOQoL-BREF score below 59 points (β = 1.255; *p* = 0.035) and pain limiting the patient’s social life rated at 4 or more on the VAS (β = 1.564; *p* = 0.030). Absence of comorbidities was a negative predictor, i.e., it was associated with a lower incidence of sexual dysfunction (β = −1.030; *p* = 0.043).

OR analysis showed that patients with reduced QoL and patients with pain limiting their social life were at a 3.5 and 4.8 times higher risk of sexual dysfunction than other patients, respectively. In turn, patients with no comorbidities were 2.8 times more likely to experience no sexual dysfunction than those who had other chronic diseases beside RA.

A model that estimates the probability of frequent occurrence of sexual dysfunction systems has a logit form:

Logit P{*Y* = 1|*X*} = −2.56 + 1.03 × (No comorbidities) + 1.26 × (WHOQOL-BREF <59 pts) + 1.56 × (Pain limiting my social life ≥4 pts)(1)

The model correctly classifies 75.9% of patients.

## 4. Discussion

Sexual dysfunction is commonly associated with pathologies of other organs and systems and may directly or indirectly result from them [11,15]. Indirect contributors to sexual dysfunction include poorer QoL, lower self-esteem and confidence, depression and anxiety, and limitations in or avoidance of sexual intercourse due to fear of pain or failure [11,12,13,14]. Other reported causes of sexual dysfunction include complications of diabetes, hormonal disorders and hormonal imbalance causing sexual interest or arousal disorders, and adverse effects of medication, especially with polypharmacy [11].

Any direct association between disease and sexual dysfunctions depends on the severity of symptoms and limitations caused by the disease and on the treatment used. In the present study, the absence of comorbidities was a statistically significant independent determinant of lower sexual dysfunction incidence in RA patients, which corroborates reports published by other authors [8]. Notably, in the present RA patient group, most common RA comorbidities included hypertension and ischemic heart disease. The association between these conditions and sexual dysfunctions has been documented in the cardiology literature.

Links between chronic disease, especially cardiovascular, and sexual dysfunction in RA patients, including erectile dysfunction in men, and problems with orgasm, arousal, and satisfaction in women, was documented by Miedany [27].

In cases of polypharmacy, when RA drugs are combined with cardiovascular medication, a negative impact of certain β-blockers on arousal and erectile function is to be expected, while patients treated with nitrates cannot use phosphodiesterase type 5 inhibitors (PDE5i) for their sexual dysfunctions. In the present study, male patients most commonly reported erectile dysfunction before and during intercourse, in line with the report by Nascimento [28]. Cardiac and vascular health affects genital perfusion and blood flow through the vaginal and penile arteries. The arousal phase in men involves increased blood flow to the dilating arterial vasculature of the penis, resulting in erection. Likewise, in women, arousal is associated with increased blood flow to the labia and vasodilation in the clitoris, causing clitoral erection and increased vaginal lubrication. As arousal is the initial phase of the sexual response, its absence may prevent the progression to subsequent phases. All changes in blood flow caused by a narrowing of the blood vessels supplying the sex organs have a particularly negative impact on the arousal phase [28]. Mons et al. reported that in patients with ischemic heart disease (IHD), erectile dysfunction is not necessarily associated with full-blown, symptomatic disease; 46% of men with IHD experience erectile dysfunction, 75% of these patients have problems with achieving erection sufficient for penetration, and 67% have problems with maintaining erection [29]. Importantly, sexual dysfunction often undermines the relationship with the partner, and may lead to loss of social contact, isolation, and loneliness, all of which may be associated with a higher risk of cardiovascular disease [28]. In the present study, most patients were in relationships, but some had not had sexual intercourse with their partner in the previous 6 months. Unfortunately, the study did not include information about the reasons for discontinuing sexual activity.

Pain is another significant independent determinant of sexual dysfunction. In a study by Miedany et al., RA-associated pain was reported as a factor adversely affecting sexual function in both women and men [27]. The present findings are consistent with reports by other authors. In the present comparative analyses, statistically significant differences in terms of pain severity were found between groups broken down by the frequency of sexual dysfunction symptoms. Thus, patients with more sexual dysfunction symptoms were more likely to report pain that restricted their mobility, social life, and sleep than those with fewer or no sexual dysfunction symptoms. OR analysis showed that patients with these kinds of pain are at a considerably higher risk of sexual dysfunction. In multivariate analysis, pain limiting the patient’s social life was a statistically significant independent determinant of more severe sexual dysfunction. In a study by Fazaa Alia et al. of a group of RA patients, pain was the only independent determinant of sexual dysfunction [15]. It is worth noting that the authors emphasized the significance of physical activity for the incidence of sexual dysfunction in this patient group. This is in line with the present findings, where single-factor analysis demonstrated a positive correlation between subjectively reported reduced mobility and sexual dysfunction. As explained by Santos-Moreno, inactivity strongly affects the function of the musculoskeletal system, which includes poorer function of the muscles in the female and male sex organs, as well as poorer functioning and increased fatigability of joints during sexual activity [1]. Mobility and physical activity are linked to pain and disease activity, two factors that significantly contributed to sexual dysfunction in the present study. Disease activity and pain severity have previously been found to be associated with sexual dysfunction in RA patients [30,31], as well as with physical fitness, and especially with muscular strength [32].

The available studies demonstrate that psychosocial factors such as chronic stress, anxiety, depression, negative attitudes, loneliness, and loss of social contacts may not only result from sexual dysfunction, but also contribute to its further exacerbation. One factor contributing to sexual dysfunction is depression, which may interfere with arousal and reduce sex drive and sexual satisfaction, leading to impotence in men and low libido or anorgasmia in women [15,27,33]. Unsatisfactory sexual performance or reduced sexual satisfaction may exacerbate depression, especially in young, professionally active people who have a partner. Research shows that in men with depression, the prevalence of erectile dysfunction may reach 100%. There is evidence of a two-way association between sexual dysfunction and depression, and the presence of one may cause or complicate the other [33,34]. On the other hand, treatment of one of these disorders has been shown to alleviate the other as well. The link between chronic disease and sexual dysfunction particularly affects the area of emotion. Experiencing difficult emotions increases the probability of sexual dysfunctions. The latter may in turn have a number of consequences. For instance, they adversely affect the quality of patients’ close personal relationships, depriving them of the benefits of social support, which is extremely important in any disease. Research findings indicate that a better sex life positively affects patients’ QoL and reduces their anxiety and depression scores. The association between emotional disorders and somatic disease is also known to be significant and bidirectional. Hence, addressing sexual dysfunction has a positive impact on somatic disease treatment and vice versa [35]. Reduced sexual enjoyment or absence thereof may cause dissatisfaction, frustration, and feelings of inadequacy in the intimate relationships of both men and women. This may undermine self-confidence and cause interpersonal difficulties, thus affecting the QoL of the patient and those around them. A proportionate relationship often exists between personal satisfaction and one’s sex life: a low level of sexual satisfaction is reflected in less satisfaction with life and vice versa [36]. Findings from the present study are similar, since multivariate analysis identified QoL as a significant determinant of sexual dysfunction. In our study, poorer QoL was associated with a 3.5 times higher risk of sexual dysfunction.

Other authors report that patients who do not undergo treatment for sexual dysfunctions are also less likely to have their somatic diseases treated [35,37,38]. One could therefore venture that the consequences of sexual dysfunction are greater for chronically ill patients than for healthy individuals, hence the importance of efforts to improve functioning in this domain in patients with chronic disease [37,38]. Better diagnostics in terms of sexual health seem particularly warranted [35]. Regardless of etiology, psychotherapy and counseling may have a positive impact especially in men with sexual dysfunction. Their sexual functioning is most commonly affected by impaired self-esteem, reduced sexual satisfaction, greater difficulty in interpersonal relations, and overall QoL deterioration [38,39]. Behavioral interventions may be most beneficial when the fundamental contributors to the dysfunction are psychological, but not as much when the problems mainly result from the underlying disease. Low sex drive, no or delayed orgasm, or erectile dysfunction in men still cannot be fully cured with pharmacotherapy alone. Research confirms that causes of sexual dysfunctions include anxiety, affective disorders, or personality disorders that are left undiagnosed and untreated. A combination of psychosocial and pharmaceutical interventions may be more effective than a single type of intervention. Problems associated with sexual dysfunction are often complex and contributed to by multiple psychosocial factors. The treatment process should include all the factors that have contributed to the development and persistence of sex and relationship problems. This requires a comprehensive somatic and psychosocial assessment to identify the predisposing, precipitating, and perpetuating factors behind the dysfunction. There is a need for interdisciplinary approaches to sexual dysfunction assessment and treatment, as well as to educational interventions.

Psychological adjustment, such as disease acceptance, increases the quality of all aspects of sexual function in chronically ill patients [40,41]. The literature lacks studies on the impact of sexual dysfunctions on disease acceptance in rheumatology patients. To the best of our knowledge, the present study is the first to evaluate the association of sexual dysfunctions with disease acceptance of RA patients. However, in a study conducted on a population of patients with type 2 diabetes, sexual dysfunction has been shown to correlate with the presence of depression and disease acceptance [40]. Similarly, in a study conducted on patients with heart failure, acceptance of the disease was positively related to sexual need, frequency of intercourse, and position and technique [41].

## 5. Conclusions

Sexual dysfunction is a problem found both in women and men with RA. The most common problems include lack of orgasm and vaginal dryness in women, and erectile dysfunction in men.

Clinical factors aggravating sexual dysfunctions include pain that limits patients’ social life, mobility, and night rest; restricted mobility; and high disease activity (DAS-28). Psychological factors that contribute to sexual dysfunction include low or no illness acceptance and poor QoL.

The absence of comorbidities is an independent determinant of lower sexual dysfunction incidence, whereas low QoL and pain limiting the patient’s social life are independent determinants of increased incidence of sexual dysfunction in both sexes.

### Study Limitations

Our study had a number of limitations. Firstly, the relatively small size of the sample, recruited from a single center, may potentially have resulted in underpowered statistical analyses, especially when the sample was broken down into two groups for comparative analyses. Importantly, the study included patients treated with biological agents, which may have affected their clinical parameters and the reported pain. Another limitation is the lack of a psychological assessment of anxiety and depression in the group, both of which could significantly affect the findings. One of the limitations of the study is the failure to determine to what extent the occurrence of this type of dysfunction is specific to patients with RA compared to the general population. A limitation of the study regarding its reliability was the need to give the questionnaire to the nurses or physicians in person; since the questionnaire deals with private matters, the participants’ responses may have been influenced by social desirability bias. The next steps should involve planning a study including an assessment of RA patients’ psychological condition and the dynamics of sexual dysfunction incidence over the course of treatment.

## Figures and Tables

**Table 1:** Demographic characteristics of the 171 patients treated for RA.

Feature (Variable)	*N* (%)
Gender:	
Women, *n* (%)	95 (55.6)
Men, *n* (%)	76 (44.4)
Age (years):	
Mean ± SD	48.3 ± 14.6
Me [Q1; Q3]	48 [37; 61]
Min–Max	20–92
BMI (kg/m^2^); Mean ± SD	25.4 ± 4.4
Education:	
Basic, *n* (%)	2 (1.2)
Vocational, *n* (%)	25 (14.6)
Secondary, *n* (%)	68 (39.8)
Higher, *n* (%)	76 (44.4)
Professional activity:	
Does not work, *n* (%)	19 (11.1)
Works, *n* (%)	114 (66.7)
Pension, *n* (%)	15 (8.8)
Retired, *n* (%)	26 (15.2)
Financial standing:	
Bad, *n* (%)	4 (2.3)
Medium, *n* (%)	74 (43.3)
Good, *n* (%)	91 (53.2)
Very good, *n* (%)	2 (1.2)
Duration of RA (years); Mean ± SD	13 ± 9
Subjective assessment of mobility (pts); Mean ± SD	6.2 ± 1.6
Comorbidities:	
Hypertension, *n* (%)	46 (26.9)
Obesity, *n* (%)	1 (0.6)
Osteoporosis, *n* (%)	4 (2.3)
Ischemic heart diseases, *n* (%)	8 (4.7)
Bronchial asthma, *n* (%)	7 (4.1)
Diabetes, *n* (%)	3 (1.8)
Psoriasis, *n* (%)	3 (1.8)
Diseases of the thyroid gland, *n* (%)	12 (7.0)
Number of comorbidities:	
0, *n* (%)	108 (63.1)
1, *n* (%)	46 (26.9)
2, *n* (%)	14 (8.2)
3, *n* (%)	1 (0.6)
4, *n* (%)	2 (1.2)
Acceptance of the disease AIS (pts); Mean ± SD	29.6 ± 11.6
Disease acceptance level (AIS)	
Not accepting the disease (8–18 pts)	44 (25.7)
Average level of disease acceptance (19–29 pts)	15 (8.8)
Good level of disease acceptance (30–40 pts)	112 (65.5)
DAS-28 (pts); Mean ± SD	4.0 ± 1.9

Me—median, SD—standard deviation; BMI—Body Mass Index; AIS—Acceptance of Illness Scale; DAS-28—Disease Activity Score.

**Table 2:** Number (percentage) of responses to the sexological questionnaire on sexual dysfunction provided by RA patients.

	All *N* = 171	Women *N* = 95	Men *N* = 76	*p*
A1. Have you had a partner in the last 6 months?				0.795
1. Yes	138 (80.7)	76 (80.0)	62 (81.6)	
2. No	33 (19.3)	19 (20.0)	14 (18.4)	
A2. If so, have you lived with your partner?				0.323
1. Yes	133 (97.1)	75 (98.7)	58 (95.1)	
2. No	4 (2.9)	1 (1.3)	3 (4.9)	
B1. Reduction of sexual needs				
1. It has always occurred	34 (24.8)	21 (27.6)	13 (21.3)	0.821
2. It happened frequently	12 (8.8)	7 (9.2)	5 (8.2)	
3. It happened sometimes	52 (38.0)	28 (36.8)	24 (39.3)	
4. It never occurred	39 (28.5)	20 (26.3)	19 (31.1)	
B2. Reluctance (fear) of sexual intercourse				
1. It has always occurred	2 (1.5)	0 (0.0)	2 (3.3)	
2. It happened frequently	70 (51.1)	36 (47.4)	34 (55.7)	0.106
3. It happened sometimes	11 (8.0)	9 (11.8)	2 (3.3)	
4. It never occurred	54 (39.4)	31 (40.8)	23 (37.7)	
B3. No pleasure during sexual intercourse				
1. It has always occurred	1 (0.7)	1 (1.3)	0 (0.0)	0.666
2. It happened frequently	28 (20.4)	17 (22.4)	11 (18.0)	
3. It happened sometimes	53 (38.7)	27 (35.5)	26 (42.6)	
4. It never occurred	55 (40.1)	31 (40.8)	24 (39.3)	
B4. Difficulty getting an erection (erection) before intercourse				
1. It has always occurred	17 (12.4)	-	17 (27.9)	
2. It happened frequently	9 (6.6)	-	9 (14.8)	-
3. It happened sometimes	4 (2.9)	-	4 (6.6)	
4. It never occurred	31 (22.6)	-	31 (50.8)	
B5. Difficulty maintaining an erection during intercourse				
1. It has always occurred	15 (10.9)	-	15 (10.9)	
2. It happened frequently	6 (4.4)	-	6 (4.4)	-
3. It happened sometimes	18 (13.1)	-	18 (13.1)	
4. It never occurred	22 (16.1)	-	22 (16.1)	
B6. Vaginal dryness				
1. It has always occurred	3 (2.2)	3 (3.9)	-	
2. It happened frequently	10 (7.3)	10 (13.2)	-	
3. It happened sometimes	39 (28.5)	39 (51.3)	-	-
4. It never occurred	24 (17.5)	24 (31.6)	-	
B7. No orgasm				
1. It has always occurred	1 (0.7)	0 (0.0)	1 (1.6)	0.026 *
2. It happened frequently	7 (5.1)	7 (9.2)	0 (0.0)	
3. It happened sometimes	29 (21.2)	19 (25.0)	10 (16.4)	
4. It never occurred	100 (73.0)	50 (65.8)	50 (82.0)	
B8. Significant orgasm delay compared to previous experiences				
2. It happened frequently	5 (3.6)	5 (6.6)	0 (0.0)	0.084
3. It happened sometimes	26 (19.0)	16 (21.1)	10 (16.4)	
4. It never occurred	106 (77.4)	55 (72.4)	51 (83.6)	
B9. Premature ejaculation				
3. It happened sometimes	12 (8.8)	-	12 (19.7)	-
4. It never occurred	49 (35.8)	-	49 (80.3)	
B10. Vaginal spasm that prevents intercourse or causes pain when trying to have intercourse				
2. It happened frequently	7 (5.1)	7 (9.2)	-	
3. It happened sometimes	6 (4.4)	6 (7.9)	-	-
4. It never occurred	63 (46.0)	63 (82.9)	-	
B11. Pain during intercourse (except for pain from infections and other diseases)				
3. It happened sometimes	26 (19.0)	17 (22.4)	9 (14.8)	0.363
4. It never occurred	111 (81.0)	59 (77.6)	52 (85.2)	
B12. Excessive sexual desire (in relation to medium intensity)				0.638
2. It happened frequently	1 (0.7)	1 (1.3)	0 (0.0)	
3. It happened sometimes	34 (24.8)	18 (23.7)	16 (26.2)	
4. It never occurred	102 (74.5)	57 (75.0)	45 (73.8)	
The frequency of symptoms of sexual dysfunction:				
Always—1 pts, *n* (%)	25 (3.7)	48 (7.9)		0.002 *
Often—2 pts, *n* (%)	90 (13.1)	65 (10.6)		
Sometimes—3 pts, *n* (%)	179 (26.2)	131 (21.5)		
Never—4 pts, *n* (%)	390 (57.0)	366 (60.0)		
**Sexual dysfunction, median [Q1; Q3]**	3.3 [3.1; 3.8]	3.4 [2.9; 3.9]		0.682
The frequency of gender identity disorders:				
Often—2 pts, *n* (%)	1 (1.3)	0 (0.0)		0.653
Sometimes—3 pts, *n* (%)	2 (0.7)	3 (2.5)		
Never—4 pts, *n* (%)	149 (98.0)	119 (97.5)		
**Gender identity disorder, median [Q1; Q3]**	4.0 [4.0; 4.0]	4.0 [4.0; 4.0]		0.802
The frequency of symptoms of abnormal sexual preferences:				
Sometimes—3 pts, *n* (%)	4 (0.4)	3 (0.4)		0.590
Never—4 pts, *n* (%)	984 (99.6)	790 (99.6)		
**Abnormal sexual preferences, median [Q1; Q3]**	4.0 [4.0; 4.0]	4.0 [4.0; 4.0]		
The frequency of symptoms of sexual orientation disorders:				1.000
Never—4 pts, *n* (%)	76 (100.0)	61 (100.0)		
**Sexual orientation disorders**	4.0 [4.0; 4.0]	4.0 [4.0; 4.0]		1.000

* Statistically significant.

**Table 3:** QoL, AIS, and DAS-28 comparison between the sexes in the studied group of RA patients.

Parameter	Women *N* =76	Men *N* = 61	*p*-Value
WHOQOL-BREF	64.2 ± 9.4	64.8 ± 7.9	0.698
Physical health	45.1 ± 13.8	46.9 ± 12.6	0.448
Psychological	75.5 ± 11.1	76.7 ± 10.9	0.550
Social relationships	67.8 ± 15.2	66.9 ± 12.2	0.678
Environment	68.2 ± 12.6	68.7 ± 9.4	0.807
Overall assessment of the quality of life	3.37 ± 0.73	3.44 ± 0.76	0.563
General assessment of your own health	2.79 ± 1.04	2.54 ± 0.92	0.146
Disease acceptance level (AIS):			
Not accepting the disease (8–18 pts)	16 (21.1)	13 (21.3)	0.110
Average level of disease acceptance (19–29 pts)	8 (10.5)	1 (1.6)	
Good level of disease acceptance (30–40 pts)	52 (68.4)	47 (77.0)	
Disease Activity Score-28 for RA			
Low disease activity (0.0–4.0 pts)	49 (64.5)	45 (73.8)	0.208
Moderate disease activity (4.1–5.0 pts)	6 (7.9)	1 (1.6)	
High disease activity (5.1–10.0 pts)	21 (27.6)	15 (24.6)	
VAS: Me ± SD	6.13 ± 1.73	5.84 ± 1.66	0.113

VAS—the visual analogue scale, RA—rheumatoid arthritis; AIS—Acceptance of Illness; Me—median, SD—standard deviation.

**Table 4:** Number (percentage) of patients with RA in groups differing in the prevalence of sexual dysfunction as well as socio-demographic and clinical factors, and the results of the independence test and odds ratios with 95% confidence intervals.

	Declaring Sexual Dysfunction		*p*-Value	OR (95% CI)
	Frequent *N* = 90	Rare 47		
Gender:				
Women, *n* (%)	52 (57.8)	24 (51.1)	0.453	1.31 (0.65–2.66)
Men, *n* (%)	38 (42.2)	23 (48.9)		1.00 (ref.)
Age:				
≥38 years, *n* (%)	65 (72.2)	28 (59.6)	0.133	1.76 (0.84–3.71)
<38 years, *n* (%)	25 (27.8)	19 (40.4)		1.00 (ref.)
BMI:				
≥24.2 kg/m^2^, *n* (%)	47 (52.2)	22 (46.8)	0.547	1.24 (0.61–2.52)
<24.2 kg/m^2^, *n* (%)	43 (47.8)	25 (53.2)		1.00 (ref.)
Marital status:				
Single, *n* (%)	23 (25.6)	8 (17.0)	0.358	1.67 (0.68–1.10)
In relationship, *n* (%)	67 (74.4)	39 (83.0)		1.00 (ref.)
Education:				
Below higher education, *n* (%)	51 (56.7)	20 (42.6)	0.117	1.77 (0.87–3.60)
Higher, *n* (%)	39 (43.3)	27 (57.4)		1.00 (ref.)
Financial standing:				
Good or very good	54 (60.0)	27 (57.4)	0.773	1.11 (0.54–2.27)
Bad or medium	36 (40.0)	20 (42.6)		1.00 (ref.)
Professional activity				
Yes, *n* (%)	65 (72.2)	31 (66.0)	0.447	1.34 (0.63–2.87)
No, *n* (%)	25 (27.8)	16 (34.0)		
Duration of RA				
<11 years	45 (50.0)	19 (40.4)	0.286	1.47 (0.72–3.01)
≥11 years	45 (50.0)	28 (59.6)		1.00 (ref.)
Subjective assessment of mobility				
<6 pts	36 (40.0)	9 (19.2)	0.023	2.81 (1.22–6.52) *
≥6 pts	54 (60.0)	38 (80.8)		1.00 (ref.)
Number of comorbidities				
<1	65 (72.2)	28 (59.6)	0.132	1.76 (0.84–3.71)
≥1	25 (27.8)	19 (40.4)		1.00 (ref.)
AIS (pts)				
<26 pts	28 (31.1)	4 (8.5)	0.003	4.85 (1.59–14.8) *
≥26 pts	62 (68.9)	43 (91.5)		1.00 (ref.)
WHOQOL-BREF (pts)				
<59 pts	27 (30.0)	6 (12.8)	0.042	2.83 (1.11–7.71) *
≥59 pts	63 (70.0)	41 (87.2)		
DAS-28 (pts)				
≥3.5 pts	37 (41.1)	10 (21.3)	0.020	2.58 (1.14–5.84) *
<3.5 pts	53 (58.9)	37 (78.7)		

BMI—Body Mass Index; RA—rheumatoid arthritis; AIS—Acceptance of Illness Scale; DAS-28—Disease Activity Score; *—*p* < 0.05.

**Table 5:** Number (percentage) of RA patients in groups differing in the prevalence of sexual dysfunction and pain levels on the visual analogue scale (VAS), as well as the results of the independence test and odds ratios with 95% confidence intervals.

Influence of Perceived Pain on Aspects of Quality of Life (VAS)	Declaring Sexual Dysfunction		*p*-Value	OR (95% CI)
	Frequent *N* = 90	Rare 47		
Pain limited my daily activities ≥4	65 (72.2)	28 (59.6)	0.132	1.76 (0.84–3.71)
Pain limited my ability to move ≥3	62 (68.9)	23 (48.9)	0.022	2.31 (1.12–4.77) *
Pain making it impossible to work ≥4	57 (63.3)	22 (46.8)	0.063	1.96 (0.96–4.01)
Pain affecting my mood ≥5	60 (66.7)	27 (57.5)	0.287	1.48 (0.72–3.06)
Pain limiting my social life ≥4	56 (62.2)	16 (34.0)	0.002	3.19 (1.52–6.68) *
Because of the pain I sleep badly at night	49 (54.4)	20 (42.6)	0.186	1.61 (0.79–3.29)
I have trouble falling asleep	24 (26.7)	18 (38.3)	0.161	0.59 (0.28–1.24)
Pain wakes me up at night	29 (32.2)	18 (38.3)	0.477	0.77 (0.37–1.60)
I wake up for a reason other than pain	30 (33.3)	12 (25.5)	0.347	1.46 (0.66–3.21)
Number of hours slept ≥6	72 (80.0)	30 (63.8)	0.039	2.27 (1.03–4.98) *

VAS—the visual analogue scale, *—*p* < 0.05.

**Table 6:** Number (percentage) of RA patients in groups differing in the prevalence of sexual dysfunction and pain levels on the visual analogue scale (VAS), as well as the results of the independence test and odds ratios with 95% confidence intervals.

	Multivariate Logistic Regression		
	B	*p*	OR (95% CI)
Age ≥38 years	0.633	0.231	1.88 (0.67–5.33)
Education level below higher	0.685	0.134	1.99 (0.81–4.88)
No comorbidities	−1.030	0.043	2.80 (1.04–7.59) *
Subjective assessment of mobility <6 pts	0.178	0.787	1.20 (0.32–4.41)
AIS <26 pts	0.946	0.291	2.58 (0.44–15.1)
WHOQOL-BREF <59 pts	1.255	0.035	3.51 (1.09–11.3) *
DAS-28 ≥3.5 pts	−0.090	0.898	0.91 (0.23–3.61)
Pain limited my ability to move ≥3	−0.125	0.861	0.88 (0.22–3.60)
Pain limiting my social life ≥4	1.564	0.030	4.78 (1.17–19.5) *
Number of hours slept ≥6	0.898	0.058	2.45 (0.97–6.21)

AIS—Acceptance of Illness Scale; DAS-28—Disease Activity Score; Chi-squared = 35.6, df = 10, *—*p* < 0.001.

## Data Availability

Data is available upon reasonable request. The data can be obtained from the corresponding author upon request after obtaining appropriate ethical approval.
